# pH/NIR Light-Controlled Multidrug Release via a Mussel-Inspired Nanocomposite Hydrogel for Chemo-Photothermal Cancer Therapy

**DOI:** 10.1038/srep33594

**Published:** 2016-09-20

**Authors:** Amin GhavamiNejad, Melisa SamariKhalaj, Ludwig Erik Aguilar, Chan Hee Park, Cheol Sang Kim

**Affiliations:** 1 Department of Bionanosystem Engineering Graduate School, Chonbuk National University, Jeonju City, Republic of Korea; 2 Division of Mechanical Design Engineering, Chonbuk National University, Jeonju City, Republic of Korea

## Abstract

This study reports on an intelligent composite hydrogel with both pH-dependent drug release in a cancer environment and heat generation based on NIR laser exposure, for the combined application of photothermal therapy (PTT) and multidrug chemotherapy. For the first time in the literature, Dopamine nanoparticle (DP) was incorporated as a highly effective photothermal agent as well as anticancer drug, bortezomib (BTZ) carrier inside a stimuli responsive pNIPAAm-co-pAAm hydrogel. When light is applied to the composite hydrogel, DP nanoparticle absorbs the light, which is dissipated locally as heat to impact cancer cells via hyperthermia. On the other hand, facile release of the anticancer drug BTZ from the surface of DP encapsulated hydrogel could be achieved due to the dissociation between catechol groups of DP and the boronic acid functionality of BTZ in typical acidic cancer environment. In order to increase the synergistic effect by dual drug delivery, Doxorubicin (DOXO) were also loaded to pNIPAAm-co-pAAm/DP-BTZ hydrogel and the effect of monotherapy as well as combined therapy were detailed by a complete characterization. Our results suggest that these mussel inspired nanocomposite with excellent heating property and controllable multidrug release can be considered as a potential material for cancer therapy.

Photothermal therapy (PTT) utilizes photo-absorbing agents to convert light energy to heat at the target site to ablate cancer cells^1^,^2^. This technique has often been used in combination with other therapeutic approaches (i.e., chemotherapy^3^, radiotherapy^4^,^5^, and gene therapy^6^) to enhance the antitumor effects. One of the most important issues in phototherapy of cancer is the limited light penetration depth. One way to solve this issue is to shift the excitation wavelength to near infrared (NIR) area^7^. The effective penetration depth of NIR light is reported to be no higher than several centimeters^8^. For some types of cancers, such as oral cancer, skin cancers, esophageal cancer, and colon cancers phototherapy can be used with the help of appropriate facilities such as gastroscopy and endoscopy^9^. To date, substantial efforts have been made to provide an effective photo-absorbing agent; however, most of the reported photothermal agents have typically been nonbiodegradable and thus potentially toxic for bioapplication^10^. Of the various photo-absorbing agents, dopamine nanoparticles (DP) have been recognized to have excellent biocompatibility and non-cytotoxicity with strong photothermal conversion efficiency, as well as excellent dispersibility in water^11^,^12^,^13^,^14^. Furthermore, this mussel-inspired biopolymeric nanoparticle contains catechol groups with their interesting chemical properties^15^,^16^,^17^. Catechol groups are a fascinating class of ligands that can be utilized to bind and release boronate-containing anti-cancer drugs (e.g., bortezomib (BTZ)) in a pH-dependent manner^18^.

The antitumour activity of BTZ is mainly attributed to its cytostatic effects and induction of apoptosis, rather than on direct killing^19^. Thus, BTZ alone has not been effective at inhibiting many types of tumors^20^. Recently, clinical trials have suggested that BTZ in combination with other anticancer drugs (e.g., doxorubicin (DOXO)) have a synergistic activity^21^. For example, Mitsiades *et al*.^22^ have examined the combination of DOXO and BTZ in patients with advanced disease to have further increased the partial response rate significantly more than that seen with other anticancer drugs (e.g., dexamethasone or thalidomide) combined with BTZ. It also has been reported that the combination of BTZ and DOXO was super synergistic when DOXO was given prior to BTZ^22^,^23^. In summary, the controlled release of BTZ and DOXO into cancerous cells in a way such that DOXO releases before BTZ could represent a promising technique to provide effective cancer treatments.

Recently, stimuli responsive hydrogels have gained considerable attention as one of the most promising drug-delivery vehicles^24^. One of the most attractive types of stimuli responsive hydrogels for locally controlled drug release are poly(N-isopropylacrylamide) (PNIPAM) hydrogels with a lower critical solution temperature (LCST) of around 32 °C^25^. This transition temperature can be easily adjusted to be between body temperature (37 °C) and hyperthermia temperature (43 °C) by copolymerization with hydrophilic monomers such as acrylamide^26^. Therefore, such smart hydrogels could be particularly suitable when both hyperthermia and chemotherapy are required.

This study aimed to prepare a smart composite hydrogel with both pH-dependent drug release in a cancer environment and heat generation based on NIR laser exposure, for the combined application of photothermal therapy (PTT) and chemotherapy. For the first time in the litrature, DP were incorporated as a highly effective photothermal agent inside a stimuli responsive pNIPAAm-co-pAAm hydrogel. The composite hydrogels were loaded with bortezomib (BTZ) and doxorubicin (DOXO), two cancer therapeutics with differing methods of cancer-killing action. The prepared composite hydrogel exhibited an efficient heating ability under NIR laser exposure. The swelling-deswelling of the nanocomposite hydrogel, as well as releasing DOXO, can be controlled remotely by NIR laser exposure or non-exposure. Furthermore, in a typical acidic cancer environment, the conjugation between catechol and BTZ dissociates, also releasing this drug in a controllable manner. In the light of the experimental results, we conclude that our mussel-inspired nanocomposite hydrogel, with excellent heating properties and controllable multidrug release, can be considered as a potential material for cancer therapy due to the synergistic effect of PTT and chemotherapy.

## Results and Discussion

**Figure 1:**
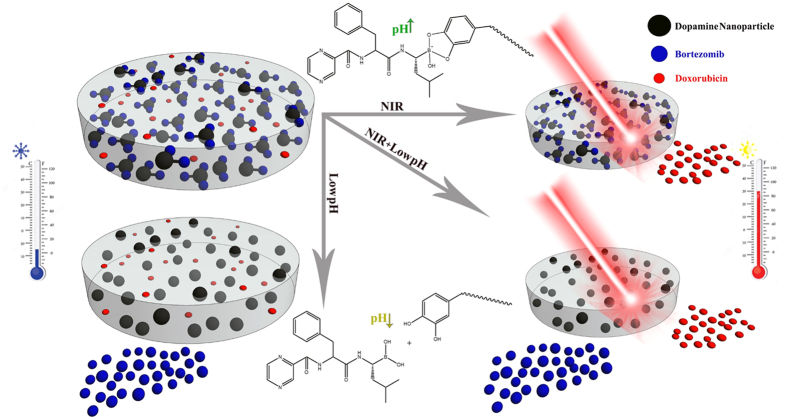
Schematic illustration of the multi-stimuli responsive mussel-inspired hybrid hydrogel as a single platform for synergistic anticancer treatment, combining both PTT and multidrug chemotherapy.

**Figure 2:**
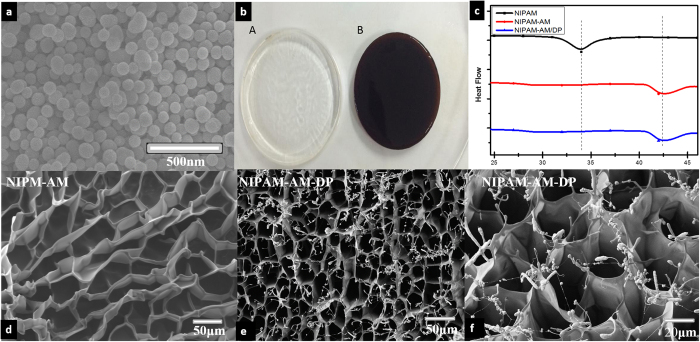
(**a**) SEM image of DP; (**b**) photographs of pure NIPAM-AM (left) and NIPAM-AM-DP prepared with 1 mg mL^−1^ DP (right); (**c**) DSC result of pure NIPAM, NIPAM-AM, and NIPAM-AM/DP hydrogels; (**d**) FESEM images of NIPAM-AM hydrogel; and (**e**,**f**) NIPAM-AM-DP hydrogel.

Figure 1 presents the schematic of multifunctional drug-loaded composite hydrogel for synergistic cancer therapy. Dopamine nanoparticles (DP) were synthesized via the self-polymerization of dopamine, according to a method that has been previously reported^11^. Scanning electron microscopy (SEM) indicated that the average diameter of the DP were approximately 70 nm (Fig. 2a). Then, the DP were dispersed in a deionized water/DMSO mixture (V/V = 10:1) and predetermined amounts of BTZ were added into the DP dispersion to start complexation between the catechol functionalities and the boronic acid active site of BTZ. It should be mentioned that the possibility of complexation between BTZ and catechol groups has been deeply studied in our previous works^17^,^27^. The resulting BTZ-loaded nanoparticles still kept the properties of DP nanoparticles, including strong photothermal conversion efficiency and excellent dispersibility in water. To combine photothermal properties and chemotherapy in a single platform, we then synthesized the pNIPAAm-co-pAAm/DP-BTZ hydrogel by incorporating DP-BTZ into the initial monomer solutions of NIPAAm and AAm, followed by radical polymerization. In order to introduce a multimodal treatment and multidrug release, we also prepared DOXO-loaded pNIPAAm-co-pAAm/DP-BTZ hydrogel by an equilibrium partitioning technique, as discussed in the experimental section.

The digital images showed the color changes of the hydrogels in the absence and presence of DP nanoparticles, evolving from transparent to brown (Fig. 2b). The LCST of the hydrogels was also studied by DSC, with results illustrated in Fig. 2c. The results indicated that the existence of only 5 mol% AAm in the structure of the hydrogels could shift the LCST of the hydrogels from 33 °C to 42 °C. Furthermore, the LCST of the hydrogel did not change in the presence of DP nanoparticles (up to 0.5 mg). Figure 2d–f show FESEM images of freeze-dried native and composite hydrogels. Both hydrogels showed high macroporosity, and their pores were interconnected to form an “open-cell” structure^28^. In the composite hydrogel samples, clusters of particles were not observed, indicating that the DP nanoparticles were uniformly dispersed into the hydrogels. An additional XRD measurement was also conducted to prove this observation (Figure SI1). After looking closely at the FESEM images of the composite, it also can be noted that surface-attached polymer chains existed on the surface of the DP nanoparticles and apparently some polymer chains grafted on the surface of the DP nanoparticles during polymerization time. This grafting formed during hydrogel preparation could provide multiple benefits for this system. For example, the strong interaction between the polymer chains and the nanoparticles could be expected to reinforce the mechanical properties of the hydrogel^29^,^30^. Furthermore, both the nanoparticles, as well as BTZ-loaded on the nanoparticles, could not escape from the polymer network during the swelling-deswelling and washing process, which could be a beneficial property for the controlled drug release. TGA measurement was performed to prove this (Figure SI2).

**Figure 3:**
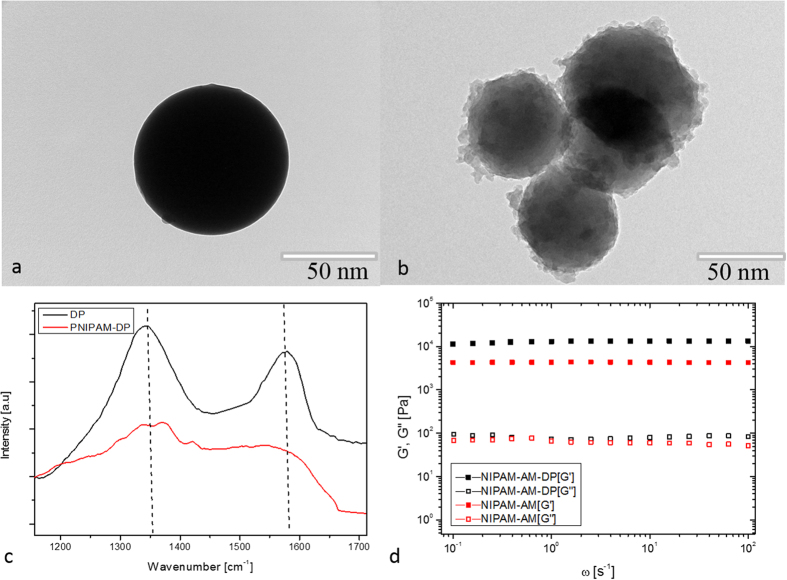
(**a**) TEM images of DP, (**b**) polyNipam grafted on the surface of DP, (**c**) Raman data of DP and polyNipam grafted on the surface of DP, and (**d**) G′ and G′′ dependency with the frequency for native (NIPAM-AM) and composite (NIPAM-AM-DP) hydrogels.

**Figure 4:**
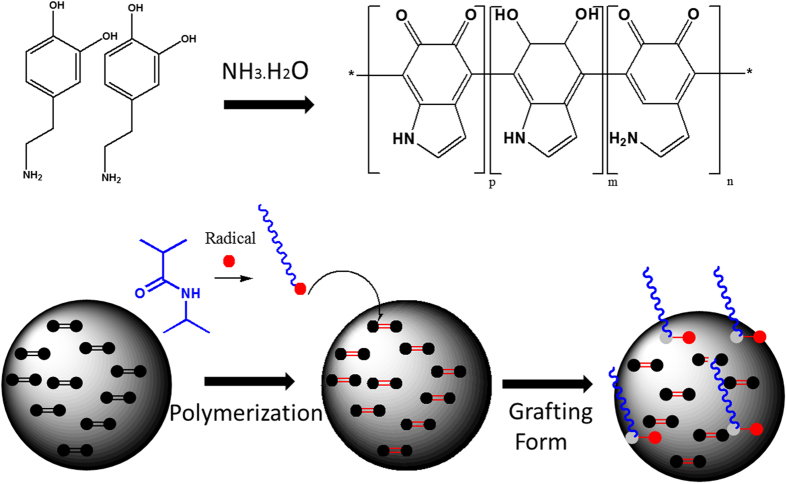
A possible structure of DP and schematic of the interactions between the polymer chains and DP.

To confirm the possibility of grafting the polymer on the DP nanoparticles during the polymerization step, control tests of *in situ* free radical polymerization were conducted between NIPAM and DP nanoparticles without the addition of cross-linker. After polymerization, the samples were purified by repeated washing, dialysis, and centrifugation to remove free PNIPAM chains. The morphological structure of DP and the PNIPAM grafted DP were examined by TEM after a washing process (Fig. 3a,b). It could be seen that the DP nanoprticles were spherical in shape and had a smooth surface. However, in Fig. 3b, it is clearly observed that, after polymerization, the PNIPAM chains existed on the surface of the DP. Unfortunately, the unknown structure of polydopamine makes it very hard to have a clear idea of the grafting mechanism^31^. It is generally accepted that DP predominantly contained residual C=C double bonds and delocalized π-electron structures^32^,^33^. Therefore it can be suggested that DP can be activated via free radical initiators to open their π-bonds or C=C double bonds then participate in the polymerization of monomers (Fig. 4)^34^. A similar strategy has been suggested by GhavamiNejad, *et al*. as well as others for covalently graft polymer chains on carbon based particles^35^,^36^. It should be also noted that, DP are likely to contain defects on the surface, which make them be able to be activated much more easily. Since the concentration of DP was very low, a large amount of PNIPAM were immobilized on the surface of the DP nanoparticles. It could also be noted that there was heterogeneity in the locations of polymer chains on the surface of the particles, as the active sites (C=C) were not homogeneously distributed on the DP nanoparticles.

In addition, the grafting of PNIPAM on DP surfaces was attested by Raman spectra (Fig. 3c). The Raman spectrum of DP nanoparticles showed two broad peaks at 1355 cm^−1^ and 1577 cm^−1^ assigned to the aromatic structure of polydopamine^37^. After polymerization with PNIPAM, the intensity of both peaks obviously decreased in comparison with those of DP, indicating a largely disordered structure for the samples obtained, owing to the formation of covalent bonds between the DP and the PNIPAM chains. This result was in agreement with Li *et al*. for polyvinylamine grafted onto polydopamine films^38^. The average size of DP were also measured by dynamic light scattering (DLS) (Figure SI3). The results show the average sizes of DP and the PNIPAM grafted DP were approximately 74.1 nm and 92.1, respectively. The hydrodynamic size of the PNIPAM grafted DP was much bigger than those determined by TEM, which may have resulted from the fact that the PNIPAM chains were highly stretched in water during DLS measurement^39^. To investigate the change of the electrokinetic surface potential of the DP nanoparticles with and without PNIPAM, the zeta potential of the DP and DP-PNI was determined. The zeta potential of DP nanoparticles was negative, with the value of −12.93 ± 1.4 mV, due to the deprotonation of catechol −OH groups on the dopamine^40^. However, after grafting, the surface charge of these nanoparticles became more negative (−27.24 ± 0.9 mV), which resulted in achieving a high stability and prevented the aggregation of the dispersed particles. To study the effects of DP nanoparticles on the mechanical properties of the samples, we performed frequency sweeps in the linear-viscoelastic regime to compare storage moduli (G′) and loss moduli (G′′) for NIPAM-AM and NIPAM-AM-DP hydrogels that contained 400 μg/ml DP (Fig. 3d). For both samples, G′ showed a more or less constant value, while G′′ was lower by a factor 100–1000 due to the elasticity of the samples. Moreover, the composite hydrogels had a significantly higher G′ than native gels, which can be interpreted as the consequence of the polymer-DP interactions, especially when considering that the composite contained only 400 μg/ml filler and that such a improvement in mechanical behavior cannot be due solely to the filler effect.

**Figure 5:**
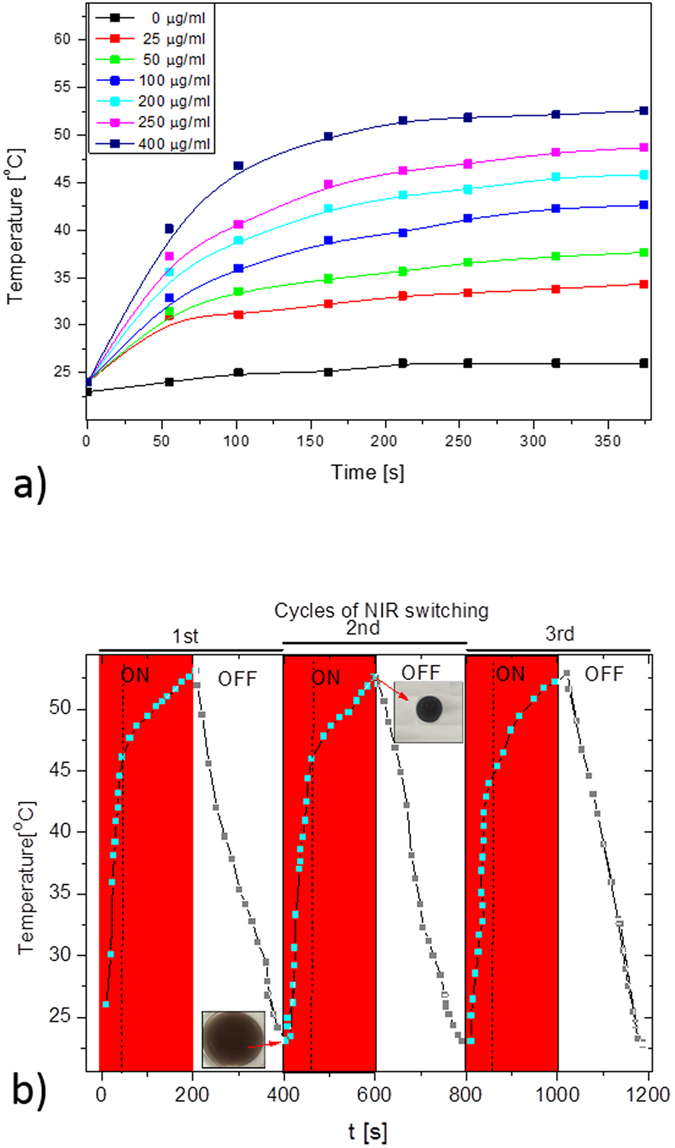
(**a**) Temperature elevation of composite hydrogels with different concentrations of DP as a function of irradiation time; (**b**) Temperature changes as a function of NIR laser on–off cycles, with the insets showing the corresponding volume of the hydrogel, and the sizes of squares being the same.

We investigated the photothermal conversion capability of DP nanoparticles and pNIPAAm-co-pAAm/DP composite hydrogels by exposing the samples to an NIR laser with a wavelength of 808 nm at a power of 2 W. As expected, we observed a significant and rapid heating ability for the samples with a higher concentration of DP. After exposing composite hydrogels that contained 400 μg/ml DP to NIR for 350 s, the surface temperature was raised from room temperature to more than 50 °C (Fig. 5a). Meanwhile, as shown in Figure SI4, the temperature elevation of 200 μg/ml of free DP nanoparticles in water was raised to 55.1 °C. This discrepancy in the heating efficiency of DP and pNIPAAm-co-pAAm/DP might be due to the existence of the grafted polymer on the surface of the nanoparticles in the composite hydrogel. Although it should also be noted that the concentration of nanoparticles loaded into this hydrogel to increase the temperature higher than 50 °C (sufficient to kill cancer cells), yet still much lower than many PTT agents encapsulated in hydrogels reported elsewhere (Supporting Information, Table S1).

The quick heating capability of the hydrogels could also be observed by an obvious rapid color change in the NIR exposed area, (Supporting Information, Video S1). In contrast, no obvious color changes occurred for the native hydrogel. The color change indicates that the temperature of the composite hydrogel was above its low critical solution transition (LCST) in the NIR exposed area, due to the photothermal effect induced by DP.

In the case of cancer therapy, a repeatable material heating property is preferred, recognizing the possibility of tumor metastasis. Most of the intravenously-injected nanoparticles for PTT and hyperthermia treatment were rapidly cleared by the reticuloendothelial system, which may seriously affect the success rate^17^,^41^. Therefore, it is highly desirable to use a nanoparticle material that is amenable to repeated heating upon laser exposure. Thus, we evaluated the repeated uniform heating and cooling profile of the pNIPAAm-co-pAAm/DP after laser exposure or non-exposure (Fig. 5b). The results indicated a uniform cyclic profile with a constant temperature rise, making it a very stable system for cancer therapy. During the laser exposure and non-exposure cycles on the small sample piece, we also observed the reversible volume changes of the hydrogel. When the NIR laser was switched on for 50 sec, the volume and weight of the hydrogel decreased by 70% and 65%, respectively, and when the NIR laser was removed, the deformed gel returned to its original shape with the same volume and weight. This controllable shrinking–swelling transition based on laser-exposure or non-exposure makes the introduced hydrogels highly promising for drug delivery applications.

**Figure 6:**
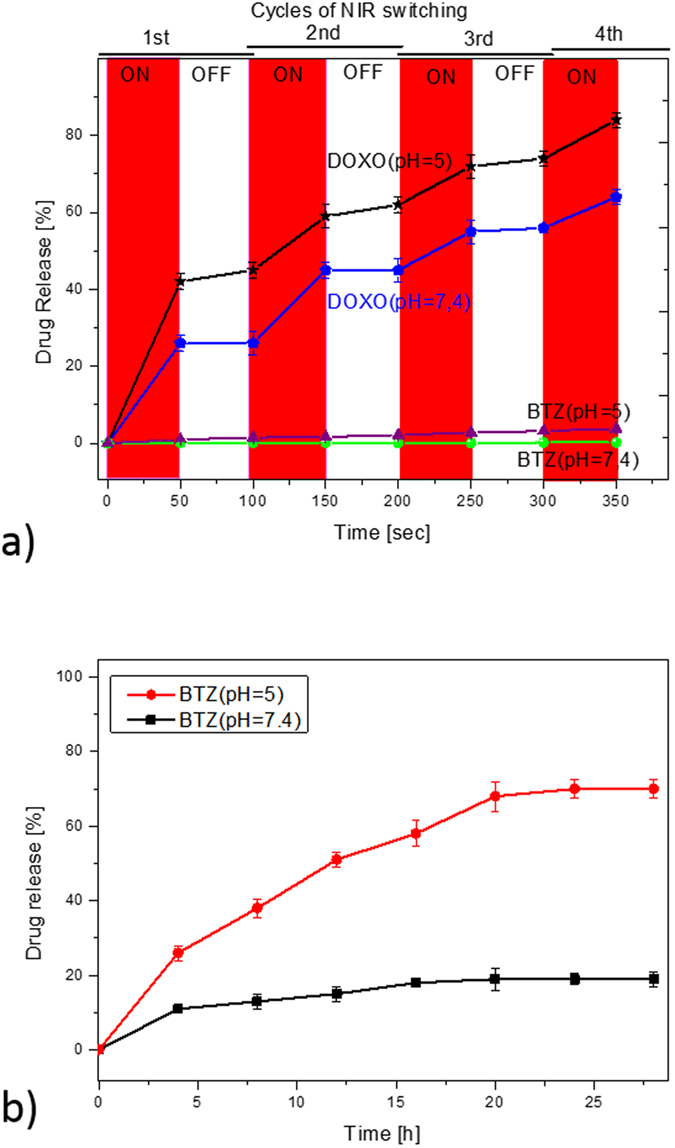
(**a**) *In vitro* drug release profiles of DOXO and BTZ from composite hydrogels in PBS at pH 5.0 and pH 7.4, with or without NIR irradiation, at 37 °C; (**b**) *In vitro* drug release profiles of BTZ from composite hydrogels, over a longer period of time.

We further evaluated the switchable changes in drug release in response to ‘on-off’ switches of NIR light in pH 7.4 and 5.0 at 37 °C. Figure 6a shows the on-demand changes in drug releasing capability in response to ‘on’ and ‘off’ switching of the NIR. As we mentioned before, after NIR exposure, a sharp shrinking process could happen, then due to the smaller size of DOXO molecules in comparison to the hydrogel network, this drug could easily run out from the hydrogel in an on-demand manner in response to temperature changes^42^. Approximately 25, 16, 11, and 7% of the loaded DOXO were released in the 1st, 2nd, 3rd, and 4th cycles of NIR exposure, respectively. This drug showed faster release at pH = 5 and about 81% of the loaded DOXO had been released to the media at the end of the 4th cycle. The faster release of DOXO in lower pH might be due to the fact that the solubility of DOXO increases as the pH value decreases^43^. Previously, such an on-demand release strategy has been used by other researchers using GO^44^, MWCNT^45^, and silica–gold^42^ encapsulated pNIPAAm-co-pAAm hydrogels. However, it should also be mentioned that not only was the concentration of filler in our work less than in other reports, but also that the concerns regarding the safety and toxicity of our PTT agent were significantly less than other agents^11^.

Compared with the release rate of DOXO, the release of BTZ was independent of NIR exposure, with negligible amounts of BTZ being released over this period. The results showed only 0.5% of the BTZ released at pH = 7.4, while nearly 3.5% released at pH = 5.0. The results also suggested that the strong complexation between BTZ and catechol groups of DP prevented the burst release of BTZ at pH = 7.4, while in an acidic environment (pH = 5), the conjugation dissociates and BTZ starts to be released and may require more time to release in higher amounts. To further assess the pH-dependent release of BTZ from the composite hydrogels, we studied the *in vitro* BTZ release over a longer period of time in Fig. 6b. The results showed that after 27 hours, 68% of BTZ released from the sample at pH = 5, but less than 20% of BTZ released at pH = 7.4, indicating the dissociation of boronic acid with catechol occurred gradually in lower pH, as suggested by previous works^17^. Consequently, under application of chemo-photothermal therapy around cancer cells (low pH), DOXO could be released prior to BTZ, which could be expected to provide a super synergistic anti-cancer effect^22^,^23^.

**Figure 7:**
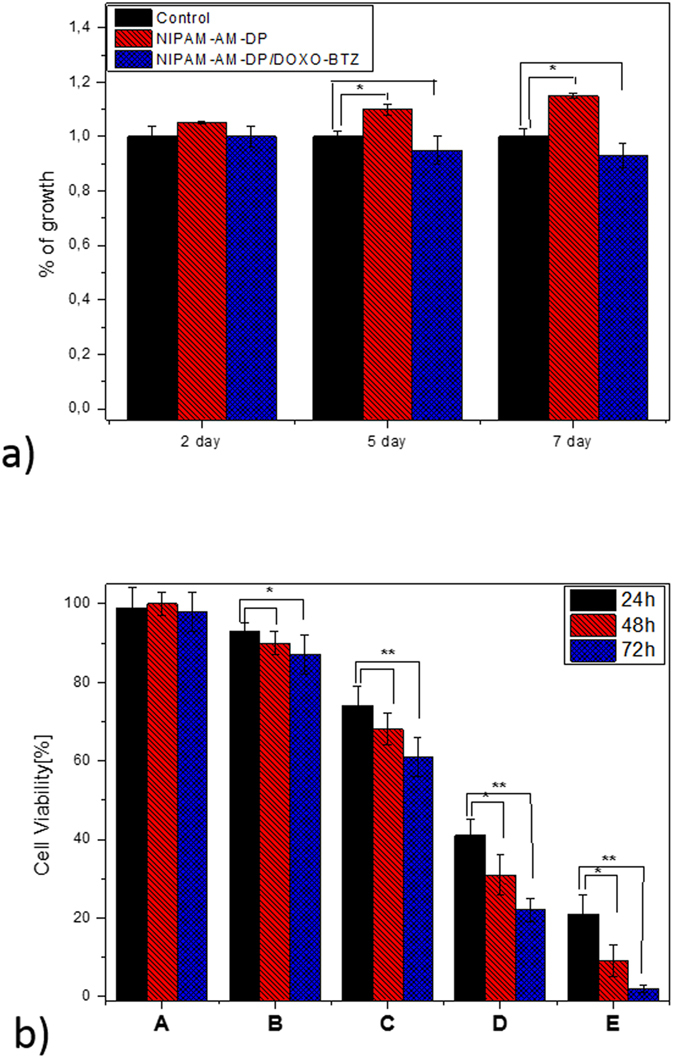
(**a**) Graph showing the MC3T3-E1 cell viability indices for control groups and therapy groups, with or without laser irradiation on 2, 5, and 7 days. The viability of the control cell was set at 100%, and viability relative to control was expressed (**b**) cell viability of CT26 colon cancer treated with (**a**) NIPAM-AM/DP (**b**) NIPAM-AM/DP+NIR, (**c**) NIPAM-AM/DP-BTZ+NIR (**d**) NIPAM-AM/DP-DOXO+NIR, and (**e**) NIPAM-AM/DP-BTZ-DOXO+NIR. *Indicates statistical significance (p < 0.05), **indicates statistical significance (p < 0.01) (one-way ANOVA post hoc Tukey test).

Previous studies have shown that the mass ratio of 1.5 to 1 for DOXO to BTZ can provide the best effect^46^. Accordingly, in the present study, the BTZ at a dose of 4 μg/mL and DOXO at a dose of 6 μg/mL were calculated to be loaded into the final hydrogels, as mentioned in the experimental section. To test the biocompatibility of the composite hydrogels, we evaluated the cytotoxicity of human cell line MC3T3-E1 cells by CCK-8 assay for 2, 5, and 7 days (Fig. 7a). We observed excellent biocompatibility for the composite hydrogel before loading the drugs, which can be attributed to the high biocompatibility of the melanine-like dopamine nanoparticle^47^. The CCK-8 assay of the sample containing drugs also showed that the mammalian cell viability was not greatly affected, due to the low concentrations of drugs.

**Figure 8:**
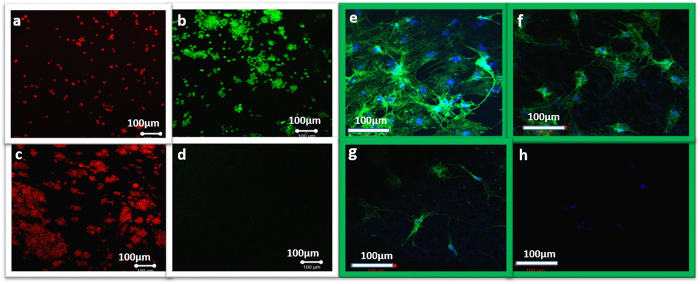
Live/dead assay displaying localized tumoricidal effects. (**a**,**b**) only photothermal therapy (NIPAM-AM/DP+NIR), and (**c**,**d**) combined application of photothermal therapy and chemotherapy (NIPAM-AM/DP-BTZ-DOXO+NIR). *In vitro* cytoskeletal imaging with Alexa Fluor^®^ 488 Phalloidin (green fluorescence): (**e**) NIPAM-AM-DP hydrogels (control), (**f**–**h**) NIPAM-AM/DP-BTZ-DOXO+NIR after 24 h/48 h/72 h of incubation. Cell nuclei were stained with DAPI (blue fluorescence).

After confirming the biocompatibility and controllable drug release, we further proceeded to evaluate the anticancer efficacy of our materials for the application of chemo-photothermal therapy. We assessed the monotherapy and combined therapy of the samples *in vitro* against CT26 colon cancer lines. These groups were treated with NIR, exposed to an 808 nm laser for 2 cycles of 50 sec on/50 sec off on day 1, day 2, and day 3. As shown in Fig. 7b, the majority of the cells were alive for the control groups; however, the percentage of viable cells was significantly changed with the highest cytotoxicity for the combined application of chemo-photothermal therapy. These results clearly demonstrated that optimum anticancer efficiency would be achieved when integrating DOXO, BTZ, and DP into a single platform. The results also supported using a live/dead assay (Fig. 8a–d). These results clearly indicated that the group containing multidrug under NIR exposure exhibited more intense red fluorescence (from dead cells) compared to control group treated with only NIR, a finding in accordance with our *in vitro* cell viability results. These findings demonstrated the introduced composite hydrogel could effectively kill the cancer cells through the multidrug chemotherapy and photothermal effects, and thus could be used as an effective method for tumor therapy. Further confirmation was carried out by performing cytoskeletal F-actin staining (Fig. 8e–h). The effect of NIPAM-AM/DP-BTZ-DOXO+NIR on the F-actin organization in the CT26 cells can be clearly visualized through the changes in the cell morphology. The changes such as membrane alteration and cytoskeletal damage including cell rounding and blebbing, suggesting that apoptosis was the cell death mechanism^48^,^49^. In the case of the combined application of chemo-photothermal therapy, many of the cells exhibited shrunken morphology with membrane blebbing on their surface and the changes were more pronounced as the incubation time increased. The steady decline in viability of the cytoskeletal morphology of the NIPAM-AM/DP-BTZ-DOXO+NIR treated cells clearly indicates that the treated cells are prone to perturbation, and thereby initiation of apoptotic signals. Therefore, we can conclude that the cytoskeletal damage caused by the NIPAM-AM/DP-BTZ-DOXO+NIR is irreversible, and the cells cannot recover their original structure.

## Conclusion

In conclusion, we reported a multi-stimuli responsive mussel-inspired hybrid hydrogel as a single platform for synergistic anticancer treatment, combining both PTT and multidrug chemotherapy. The hydrogel was composed of a highly biocompatible polydopamine nanoparticle, as a near-infrared absorbing agent embedded within stimuli-sensitive poly (NIPAAm-co-AAm) hydrogels. In order to introduce a multimodal treatment and multidrug release, BTZ and DOXO were loaded into the hydrogel through complexation with catechol groups of DP and an equilibrium partitioning technique, respectively. When light was applied to the hydrogel, DP nanoparticles absorbed the light, which was dissipated locally as heat to impact cancer cells via hyperthermia. Furthermore, the increase of temperature induced by NIR laser irradiation to more than the LCST of the hydrogel caused rapid deswelling, followed by release of DOXO in an on-demand manner. Moreover, in a typical acidic cancer environment, the conjugation between catechol and BTZ dissociates, also resulting in the controllable release of this drug. Morphological characterization showed the polymer chains were grafted on the surface of DP through covalent bonds. This strong interaction not only improved the mechanical properties of the hydrogel, but also prevented the release of the nanoparticles, as well as BTZ loaded on them, during swelling-deswelling and the washing process. Finally, *in vitro* studies confirmed that our mussel-inspired nanocomposite with excellent heating properties and controllable multidrug release can be considered as a potential material for cancer therapy, due to the synergistic effect of PTT and chemotherapy.

## Materials and Methods

### Materials

3, 4-dihydroxyphenethylamine hydrochloride (dopamine hydrochloride, Sigma-Aldrich, 99%); doxorubicin hydrochloride (Sigma-Aldrich, 99%); N,N′-methylenebis (acrylamide) (MBA, Sigma-Aldrich, 99%); ammonium peroxodisulfate (APS, Sigma-Aldrich, >98%); N,N,N′,N′-tetramethylethylenediamine (TMEDA, Fluka, >99%); bortezomib (Santa Cruz Biotechnology, USA) were used as received. N-Isopropylacrylamide (NIPAM, Sigma-Aldrich, 98%) and Acrylamide (AM, Sigma-Aldrich, 98%) were recrystallized from a 65:35 (v/v) mixture of hexane and benzene before use. All aqueous solutions were prepared with ultrapure water purified with a Milli-Q UV-Plus water purification system (Millipore, Bedford, MA).

### Synthesis of dopamine nanoparticle (DP)

DP were synthesized according to a previously reported method^11^. Ammonia aqueous solution (2 mL, NH) was added into a mixture of deionized water (90 mL) and ethanol (40 mL) under mild stirring at room temperature for 30 minutes. Dopamine hydrochloride (0.5 g) was dissolved in deionized water (10 mL) and then added into the above mixture solution. The reaction was allowed to proceed for 24 h. DP were obtained by centrifugation and washed with water three times.

### Incorporation of BTZ onto DP

For these experiments, 50 mg of DP were dispersed in a deionized water/DMSO mixture (V/V = 10:1) and sonicated for 15 min. The pH of the dispersion was adjusted to 9 with drops of NaOH 0.1 M. Then, pre-determinated amounts of BTZ were added into the DP dispersion, and the mixture was kept overnight in a shaking incubator at room temperature to initiate complexation between the catechol functionalities and the boronic acid active site of BTZ. The dispersions obtained were dialyzed against deionized water for 24 h (200 mL, the solvent was changed three times) at room temperature using a dialysis membrane of 10 kDa (MWCO) in order to eliminate the non-loaded drug. Finally, the BTZ loaded-DP dispersions were obtained by centrifugation, weighted, and stored for future utilization. The amount of drug-loaded nanoparticle was measured by capturing the UV spectra of BTZ with a previously-obtained calibration curve with a dilution series (0.1; 1; 5; 10; 15; 25; 37.5; 50; and 75 μg mL^−1^) (Figure SI5). The amount of drug incorporated onto DP was estimated to be 85% through Equation 1, an expected result due to the high number of catechol groups on the surface of DP.


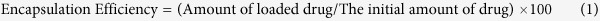


### Synthesis of composite hydrogels

NIPAM (1.06 mmol), AAM (0.056 mmol) as monomers, and MBA (0.008 gr) as a cross-linker were added to a vial containing 2 ml of water that contained known concentrations of DP or BTZ-DP at pH 9. The mixtures were stirred for 5 min, and the dissolved oxygen was removed by bubbling nitrogen through the solution for 20 min. An adequate amount of APS (20 μl) as an initiator for free radical polymerization was added to the vial under nitrogen bubbling at room temperature. After the solution was homogenized, TMEDA (20 μl) was added as an accelerator into the monomer solution to initiate radical polymerization, while the pH of the dispersion was adjusted to 9. After mild shaking for several seconds, the solution was transferred to the mold. Polymerization was continued for 6 h. After polymerization, the prepared hydrogel was removed from the mold and immersed in high pH double-distilled water at room temperature for at least 24 h, during which time the water was regularly refreshed in order to remove unreacted compounds. The amount of BTZ-incorporated composite hydrogels at the end of washing process was estimated to be around 63% of the initial concentration of BTZ.

### Field emission scanning electron microscopy (FESEM)

The surface structure and morphology of the freeze-dried hydrogels were studied via field emission scanning electron microscopy (FESEM; Zeiss Supra 40VP). To prepare freeze-dried samples, after they had been maintained in water at 15 °C for a week, the hydrogels were transferred into liquid nitrogen for 15 min, and they were then freeze-dried at −43 °C under a vacuum of 0.1 Pa for 48 h to thoroughly remove the water. The cross-sections were observed by placing the freeze-dried hydrogel samples into liquid nitrogen for a sufficient length of time, and they were then mechanically fractured and stuck to a sample holder.

### X-ray powder diffraction (XRD)

X-ray diffraction experiments were performed at room temperature to study the crystalline structure of the materials using an X-ray diffraction system (Philips X Pert-MRD, the Netherlands) employing CuKα radiation (X-ray wavelength λ = 1.5406 Å) under normal laboratory conditions. The chemical compositions and crystallographic structures of samples were recorded by varying the angle following Bragg’s law, which is satisfied by the d-spacing in polycrystalline materials.

### Raman spectroscopy

A micro-Raman spectrometer (Nanofinder 30) with an argon ion laser at an excitation wavelength of 632.8 nm and an infrared spectrometer (Tokyo Instrument, Inc.) were employed.

### Transmission electron microscopy (TEM)

A Transmission Electron Microscope (TEM, JEOL (Japan)/JEM-2010 operated at 200 KV) was used to demonstrate the morphology of the DP and PNIPAM grafted on DP particles.

### Thermogravimetric analysis (*TGA*)

TGA measurements were run on a SDT Q600 TGA/DSC system under a N2 purge from room temperature to 800 °C at a heating rate of 10 °C min^−1^.

### Dynamic Light Scattering (*DLS*)

DLS measurements were conducted at 25 °C with a DelsaMax Pro. The average value was obtained from three replicated measurements for each sample.

**Zeta potential** measurements were performed at 25 °C on the DelsaMax Pro device, using the M3-PALS technology.

### Incorporation of DOXO into the nanocomposite hydrogels

The loading of DOXO in crosslinked polymer networks can be accomplished by an equilibrium partitioning technique. In this technique, the partially swollen nanocomposite hydrogel that contains a known concentration of BTZ was put in contact with 200 μl of a different concentration of DOXO in water. The solution was absorbed almost instantaneously inside the hydrogel network. The swollen drug-loaded hydrogel was removed and cleaned with deionized water. Then the amount of DOXO loaded in the sample was determined through Equation 1. The result showed that almost the total amount of DOXO (98%) had been loaded into the hydrogel through this simple procedure.

### Preparation of PNIPAM grafted DP

PNI–DP nanocomposites were prepared via *in situ* free radical polymerization. First DP was added in 10 mL of water and sonicated for 120 min to make a homogenous brown dispersion (1 mg/mL). The NIPAM monomer was dissolved in distilled water, then the DP aqueous dispersion was gradually added to the NIPAM solution to obtain homogeneous NIPAM–DP solutions by stirring under a N2 atmosphere. Next, APS and TMEDA (10 mmol) were added to the solution under stirring. Then, free radical polymerization was allowed to proceed for 24 h. The as-prepared PNIPAM–DP suspension was homogeneous with a brown color and without precipitates. Finally the composites were washed several times by dialysis and centrifugation to remove any PNIPAM not connected to the DP particles.

### Photothermal irradiation

Hydrogels were cut into discs of diameter 10 mm and irradiated with an 808 nm laser at a power density of 2 W cm^−2^ at room temperature. The samples were heated for 200 s, and the heating characteristics were automatically recorded using type-T thermocouples and a real-time data acquisition system (NI-DAQR, National instrument, USA) with the Lab VIEW program. Before each experiment, the temperature was calibrated and stabilized for 10 min. To investigate the thermal reversibility, the swelling and deswelling transition of the pNIPAAm-co-pAAm/DP hydrogel was repeated several times with on and off cycles of the laser.

### Drug release studies

The release behavior of the loaded nanocomposite hydrogel discs was studied in centrifuge tubes containing phosphate-buffered saline (PBS) solution at different pH levels, including physiological (pH 7.4) and acidic (pH 5) conditions. For the experiment under NIR, the hydrogel disks were exposed to an 808 nm NIR laser at power densities of 2 W/cm^2^ for 50 s to increase the temperature of the hydrogel to 45 °C. The NIR was then turned ‘off,’ allowing cool down to 25 °C. This process was repeated for 4 cycles. The amount of BTZ and DOXO that was released during each cycle was quantified by comparing the UV–visible absorption spectra (HP 8453 UV–vis spectroscopy system, Germany) of drugs at wavelengths of 270 nm and 485 nm with previously-obtained calibration curves with a dilution series (Figure SI5 and 6). Finally, the amounts of released drugs were calculated using the following equation, plotting the percentage of drug released from the samples against time.


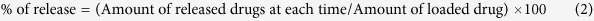


### *In vitro* biocompatibility test

The viability of the cultured cell was monitored on the second, fifth, and seventh days of culture, using a CCK (Dojindo’s cell counting kit-8) assay. Samples of the same size were sterilized under UV light and washed in phosphate buffer saline (pH 7.4). Later, samples were transferred to a 48-well plate and rinsed with medium prior to cell seeding. The fibroblast (NIH-3T3) cells were cultured at 37 °C under 5% CO_2_ in Dulbecco’s Modified Eagle Medium (DMEM, GIBCO) supplemented with 10% fetal bovine serum (FBS, GIBCO) and 1% penicillin-streptomycin. (10,000 cells/well for samples without drug content and 20,000 cells/well for samples with drug content, DMEM/high glucose supplemented with 10% FBS and 1% penicillin-streptomycin) was dispensed in a pre-incubated 48-well plate containing hydrogels and allowed to incubate in a humidified atmosphere of 5% CO_2_ at 37 °C for the designated time. Culture medium was changed every 2 days. Following the manufacturer’s instructions, 200 μL of cultured medium was transferred to a 96-well plate, while 20 μL of a CCK-8 solution was added to each well and incubated for 3 h. After 3 h of incubation, the absorbance was measured at a wavelength of 450 nm using a microplate reader (Sunrise Tecan, Austria). A standard curve was established by measuring the known number of cells prior to the experiment, and cell viability was determined from the standard curves.

### *In vitro* cancer cytotoxicity test

A cytotoxicity test was done to evaluate the effectivity of the samples in cancer apoptosis, using the CT26 colon cancer cell line (Korean Cell Line Bank, KCLB, Korea). The cells were cultured using a 24-well polystyrene dish with a cell culture media of RPMI 1640 with L-glutamine (300mg/L), 25 mM HEPES and 25 mM NaHCO_3_, 90% heat inactivated fetal bovine serum (FBS) 10% (GIBCO, USA), and 1% penicillin streptomycin (P/S, GIBCO, USA). The cells were cultured inside a 37 °C incubator with 5% CO_2_ atmosphere for 2 days. The hydrogel disks were placed into a 48-well plate and were washed three times with PBS (pH7.4) prior to cell seeding. For the samples containing drugs, we set the dose of loaded drugs to be finally released at the concentration of 6 μg/mL and 4 μg/mL for DOXO and BTZ, respectively. The cells were placed at a density of 5 × 10^4^ cells onto the hydrogels and were co-incubated at 37 °C for another 24 h. The synergistic effects of PTT and chemotherapy were evaluated in the absence and presence of a 2 W/cm^2^ 808 nm NIR. The cells were irradiated for 2 cycles of 50 sec on/50 sec off on day 1, day 2, and day 3. During NIR irradiation, the untreated coverslip was kept separately in a mini petri dish in an incubator with a humidified 5% CO_2_ environment at 37 °C, in order to rule out any environmental effect on cell death for the duration of the NIR treatment. After 24 h/48 h/72 h of incubation, the amount of viable cells was evaluated via CCK-8 assay. The cells were also chemically fixed with 4% paraformaldehyde, followed by staining with calcein and ethidium homodimer according to the manufacturer’s protocol. Finally, the stained samples, were examined using confocal laser scanning microscopy (Carl Zeiss, Japan). The cytoskeletal damage was assessed using the cytoskeletal F-actin stains Phalloidin (Alexa Fluor 488 Phalloidin, Molecular probes, USA, 488 nm, green).

### Statistical analysis

Data were compared using one-way ANOVA, in GraphPad Prism 6. Data are expressed as means ± SD of measurements (*p < 0.05 and **p < 0.01).

## Additional Information

**How to cite this article**: GhavamiNejad, A. *et al*. pH/NIR Light-Controlled Multidrug Release via a Mussel-Inspired Nanocomposite Hydrogel for Chemo-Photothermal Cancer Therapy. *Sci. Rep.* **6**, 33594; doi: 10.1038/srep33594 (2016).

## Supplementary Material

Supplementary Information

Supplementary Movie S1
