# Supplementary material for: pH/NIR Light-Controlled Multidrug Release via a Mussel-Inspired Nanocomposite Hydrogel for Chemo-Photothermal Cancer Therapy
Source: Sci Rep. 2016 Sep 20;6:33594. doi: 10.1038/srep33594 (PMC5028867; doi:10.1038/srep33594)
Supplement: Supplementary Information [file srep33594-s1.docx]

***Supporting Information***

pH/NIR Light-Controlled Multi-Drug Release via a Mussel-Inspired Nanocomposite Hydrogel for Chemo-Photothermal Cancer Therapy

*Amin GhavamiNejad^1a*^, Melisa SamariKhalaj^1a^, Aguilar Ludwig Erik, Chan Hee Park ^1,2*^ and Cheol Sang Kim ^1, 2*^*

*Department of Bionanosystem Engineering, Graduate School, Chonbuk National University, Jeonju 561-756, Republic of Korea.*

***Figure S1. XRD patterns of Dopamine nanoparticle, NIPAM-AM hydrogel and the NIPAM-AM-DP nanocomposite hydrogel***

Figure S1 shows the XRD patterns of Dopamine nanoparticle, NIPAM-AM hydrogel and the NIPAM-AM-Dopa nanocomposite hydrogel. The typical diffraction peak of dopamine nanoparticle was observed as a wide band from 2Ɵ=15-30, while the broad peak that appeared centered at 2θ=21.0 ° were ascribed to the diffraction peaks of NIPAM. However, after dopamine nanoparticle was dispersed into the PNIPAM matrix through polymerization, the XRD pattern of the freeze-dried NIPAM-AM-Dopa nanocomposite only showed the diffraction peaks of the matrix and the diffraction peak of the dopamine nanoparticle disappeared. The XRD results obviously demonstrate that the dopamine nanoparticle were dispersed well in the polymer matrix.

***Figure S2. TGA curve illustrating sample weight loss as a function of temperature.***

TGAs experiments were conducted to determine the changes in the weight percentage of DP in the hydrogels before and after NIR irradiation. As illustrated in Figure S2, pristine PDNs started to lose their weight at about 80^o^C and beyond 800^o^C, around 50% of the original DP content was left. The amount of residual mass of nanoparticle in the nanocomposite hydrogel with initial concentration of 1wt% was 0.66%, which is higher than the expected residue of 0.50% for this sample. Possibly due to the strong covalent bonding between the polymer chains and DP which can improve the thermal stability of the nanocomposite.[^35^](#_ENREF_35) It should be also mention that no changes were observed in the residual mass of the composite hydrogels with multiple swelling/deswelling transition (irradiated by NIR (3 cycles)), confirming nanoparticles were strongly interacted to the polymeric hydrogel and could not escape from the polymer network.

***Figure S3. Size distribution of DP before and after grafting measured by dynamic light scattering.***

The average size of DP nanoprticles were also measured by dynamic light scattering (DLS) in Figure 3SI. The results show the average sizes of DP and polynipam grafted DP were approximetly 74.1 nm and 92.1, respectively. The hydrodynamic size of polynipam grafted DP was much bigger than those determined by TEM, which may have resulted from the fact that the PNIPAM chains were highly stretched in water during DLS measurment. To investigate the change of the electrokinetic surface potential of the DP nanoparticles with and without PNIPAM, the zeta potential of the DP and DP-PNI was determined. The zeta potential of DP nanoparticles was negative, with the value of −12.93 ± 1.4 mV, due to the deprotonation of catechol −OH groups on the dopamine.[^1^](#_ENREF_1) However, after grafting, the surface charge of these nanoparticles became more negative (−27.24 ± 0.9 mV), which resulted in achieving a high stability and prevented the aggregation of the dispersed particles.

***Figure S4. Temperature elevation of water and dopamine nanoparticle aqueous solutions with different concentrations as a function of irradiation time.***

**Table S1** compares the heating efficiency of our NIPAM-AAM/DP hydrogels with some of the most effective composite hydrogels reported so far. The results show the photothermal conversion efficiency of our material is much higher than those of previously reported PTT agents encapsulated hydrogels.

| **Hydrogel** | **Nanoparticle/ concentration[mg/ml]** | **NIR wavelength[nm]/**  **power[W]** | **Time[s]** | **Heating ability**  **T** | **References** |
| --- | --- | --- | --- | --- | --- |
| **NIPAM-AAM** | **DP/0.4** | **808/2** | **300** | **25 to 54** | **This work** |
| **NIPAM-AAM** | **GO/1.0** | **808/2** | **300** | **25 to 52** | [**^2^**](#_ENREF_2) |
| **NIPAM-AAM** | **MWCNT/ 3.0** | **980/1.2** | **180** | **15 to38** | [**^3^**](#_ENREF_3) |
| **NIPAM** | **GO-Au/1.0** | **808/2** | **300** | **30 to 65** | [**^4^**](#_ENREF_4) |
| **NIPAM** | **Fe_3_O_4_/NG** | **808/2** | **300** | **25 to 47** | [**^5^**](#_ENREF_5) |

***Figure S5. Control graph shows the peak of BTZ with different concentrations at 270nm.***

***Figure S6. Control graph shows the peak of DOXO with different concentrations at 490nm.***

**Video is provided as follows:**

**Movie S1.** Rapid color change in the NIR exposed area of the composite hydrogel.

REFERENCES

1. Miao, Z. H.; Wang, H.; Yang, H. J.; Li, Z. L.; Zhen, L.; Xu, C. Y., Intrinsically Mn2+-Chelated Polydopamine Nanoparticles for Simultaneous Magnetic Resonance Imaging and Photothermal Ablation of Cancer Cells. *Acs Appl Mater Inter* **2015,** *7* (31), 16946-16952.

2. Zhu, C. H.; Lu, Y.; Peng, J.; Chen, J. F.; Yu, S. H., Photothermally Sensitive Poly(N-isopropylacrylamide)/Graphene Oxide Nanocomposite Hydrogels as Remote Light-Controlled Liquid Microvalves. *Adv Funct Mater* **2012,** *22* (19), 4017-4022.

3. Cheng, Z. Y.; Chai, R. T.; Ma, P. A.; Dai, Y. L.; Kang, X. J.; Lian, H. Z.; Hou, Z. Y.; Li, C. X.; Lin, J., Multiwalled Carbon Nanotubes and NaYF4:Yb3+/Er3+ Nanoparticle-Doped Bilayer Hydrogel for Concurrent NIR-Triggered Drug Release and Up-Conversion Luminescence Tagging. *Langmuir* **2013,** *29* (30), 9573-9580.

4. Cong, H. P.; Qiu, J. H.; Yu, S. H., Thermoresponsive Poly(N-isopropylacrylamide)/Graphene/Au Nanocomposite Hydrogel for Water Treatment by a Laser-Assisted Approach. *Small* **2015,** *11* (9-10), 1165-1170.

5. Zhu, C. H.; Lu, Y.; Chen, J. F.; Yu, S. H., Photothermal Poly(N-isopropylacrylamide)/Fe3O4 Nanocomposite Hydrogel as a Movable Position Heating Source under Remote Control. *Small* **2014,** *10* (14), 2796-2800.
